# A Regression-Based Approach to Diabetic Retinopathy Diagnosis Using Efficientnet

**DOI:** 10.3390/diagnostics13040774

**Published:** 2023-02-17

**Authors:** Midhula Vijayan, Venkatakrishnan S

**Affiliations:** Forus Health Private Limited, Bengaluru 560070, Karnataka, India

**Keywords:** diabetic retinopathy (DR), convolutional neural network (CNN), efficientnet, DR lesions, non-proliferative diabetic retinopathy (NPDR), proliferative diabetic retinopathy (PDR), transfer learning

## Abstract

The aim of this study is to develop a computer-assisted solution for the efficient and effective detection of diabetic retinopathy (DR), a complication of diabetes that can damage the retina and cause vision loss if not treated in a timely manner. Manually diagnosing DR through color fundus images requires a skilled clinician to spot lesions, but this can be challenging, especially in areas with a shortage of trained experts. As a result, there is a push to create computer-aided diagnosis systems for DR to help reduce the time it takes to diagnose the condition. The detection of diabetic retinopathy through automation is challenging, but convolutional neural networks (CNNs) play a vital role in achieving success. CNNs have been proven to be more effective in image classification than methods based on handcrafted features. This study proposes a CNN-based approach for the automated detection of DR using Efficientnet-B0 as the backbone network. The authors of this study take a unique approach by viewing the detection of diabetic retinopathy as a regression problem rather than a traditional multi-class classification problem. This is because the severity of DR is often rated on a continuous scale, such as the international clinical diabetic retinopathy (ICDR) scale. This continuous representation provides a more nuanced understanding of the condition, making regression a more suitable approach for DR detection compared to multi-class classification. This approach has several benefits. Firstly, it allows for more fine-grained predictions as the model can assign a value that falls between the traditional discrete labels. Secondly, it allows for better generalization. The model was tested on the APTOS and DDR datasets. The proposed model demonstrated improved efficiency and accuracy in detecting DR compared to traditional methods. This method has the potential to enhance the efficiency and accuracy of DR diagnosis, making it a valuable tool for healthcare professionals. The model has the potential to aid in the rapid and accurate diagnosis of DR, leading to the improved early detection, and management, of the disease.

## 1. Introduction

DR is the leading cause of blindness among adults worldwide [1]. DR affects an estimated 93 million people globally, and this number is likely to increase due to the rising prevalence of diabetes in countries such as China and India [2,3]. The early diagnosis and treatment of DR are critical for preventing its progression and saving patients from vision loss. However, the manual diagnosis of DR using retina fundus images by experts is a time-consuming and error-prone process, which makes it important to develop accurate and efficient computer-aided diagnosis systems.

DR can be divided into two categoris: non-proliferative diabetic retinopathy (NPDR) [4] and proliferative diabetic retinopathy (PDR) [5]. NPDR is characterized by changes in the blood vessels of the retina, while PDR is characterized by neo-vascularization [5] and is more severe than NPDR. DR can be diagnosed through an examination of the retina for the presence of specific lesions [5], including microaneurysms, haemorrhages, hard exudates, and soft exudates. The presence and characteristics of these lesions can help to classify the severity of DR.

**Figure 1:**
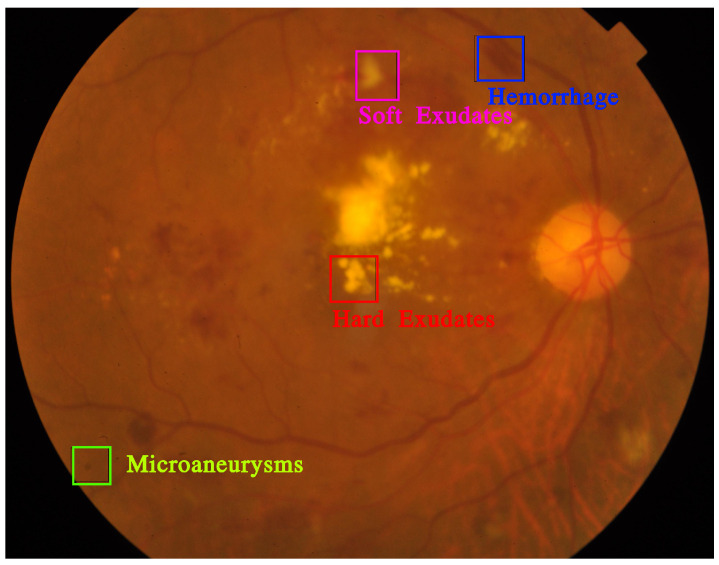
Illustration of DR lesions.

Diabetic retinopathy can be identified by the presence of certain lesions in the retina [6]. One such lesion is a microaneurysm [7], which is a small, round dot that appears on the retina in the early stages of the disease. These dots have a sharp margin and are usually no larger than 125 micrometers in size. There are six subtypes of microaneurysms, but the treatment for all of them is the same. Another type of lesion associated with diabetic retinopathy is a haemorrhage [7], which appears as a large, irregularly shaped spot on the retina and is typically larger than 125 micrometers. Hard exudates [7] are another type of lesion that result from plasma leakage and appear as yellow spots on the retina with sharp margins. Soft exudates [7], which result from nerve fiber swelling, appear as white, oval shapes on the retina. Figure 1 shows the DR lesions on fundus image.

**Figure 2:**
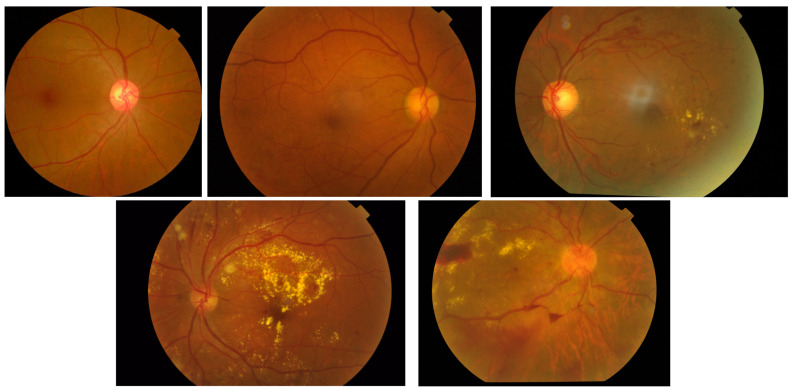
Series of DR images representing different grades are shown in order from top left to bottom right, ranging from grade 0 to grade 4.

The international clinical diabetic retinopathy disease severity scale (ICDRDSS) [8] is a classification system for diabetic retinopathy presented by the global diabetic retinopathy project group. It divides DR into five severity levels, as detailed in Table 1. This system is used to determine the appropriate course of treatment for individuals with DR based on the severity of the disease. Examples of DR images at different stages of severity are shown in Figure 2.

Each stage of diabetic retinopathy has its own unique characteristics and properties, which can be difficult for doctors to consider when making a diagnosis. According to a study by Google [9], even well-trained clinicians may struggle to accurately evaluate the stages of diabetic retinopathy manually. As a result, the idea of automatic detection has gained attention as a way to improve accuracy and efficiency in the diagnosis of DR. This paper presents a CNN-based approach for automated DR detection that has the potential to improve the speed and accuracy of diagnosis. While previous research has typically treated DR detection as a classification problem, we have instead approached it as a regression problem as the severity levels of DR follow an ordered progression and are dependent on each other. Our method shows promise in preventing vision loss in patients through the improved diagnosis of DR.

The motivation behind using a regression-based approach for DR classification lies in the fact that it provides a more interpretable way to predict the severity of DR compared to traditional classification methods. Unlike traditional classification methods, regression models can estimate the severity of DR in addition to predicting class labels, yielding a continuous output rather than a categorical one. This provides a more nuanced understanding of the progression of DR and its underlying mechanisms.

The proposed approach combines the strengths of regression and Efficientnet-B0 [10] to provide both accuracy and interpretability. By incorporating the Efficientnet-B0 architecture [10], this regression-based approach has the potential to be a valuable tool in the management of DR.

This paper is structured as follows: Section 2 explains different DR detection techniques. The presented algorithm is described in Section 3, and the results and discussions are discussed in Section 4. The paper concludes with a summary in Section 5.

## 2. Literature Review

The manual detection of DR images has been challenging because of the shortage of qualified ophthalmologists and the high cost of diagnostic procedures, especially in developing countries. Automated processing methods have been created in order to make diagnoses more accessible, rapid, and accurate. These techniques have the potential to enable early treatment and decrease the risk of blindness. Recently, machine learning models using eye fundus images have achieved high levels of accuracy in automatic DR classification. These models offer a faster and more cost-effective alternative to manual detection. There are two main categories of research in the field of DR classification [11]: traditional techniques that use handcrafted features and modern approaches that utilize deep learning. Both of these approaches will be further explored below.

Earlier automatic diabetic retinopathy classification models used a handcrafted feature-based approach, the accuracy of which was dependent on the quality of the handcrafted features. Akram et al. [12] presented a DR classification model using a hybrid structure of a support vector machine and a Gaussian mixture model. They later improved upon this method by adding additional features, as reported in a subsequent study. In [13], Adal et al. extract intensity and shape features and use these features for classification. In [14], the authors extracted texture features and trained the SVM model for the classification. A combined feature-extraction method using Haralick and ADTCWT (anisotropic dual-tree complex wavelet transform) is introduced in [15]. Feature selection can be challenging when using handcrafted features for classification. Manual feature extraction is also prone to being unreliable, leading to a shift towards the use of modern deep learning algorithms in recent papers. These algorithms offer improved robustness and accuracy, making them a popular choice in the field.

Deep learning techniques, especially CNNs, have been commonly used for the classification and identification of retinal fundus images in the detection of diabetic retinopathy. These techniques can be classified into two main categories [16]: binary DR classification and multi-level DR classification. This paper focuses on the use of CNNs for multi-level classification, and a review of recent papers is presented in Table 2. The basic steps in building a CNN-based classification model [17] typically involve creating a train and test dataset, training the CNN model, and evaluating the model.

In our analysis of the standard frameworks for diabetic retinopathy classification, we found that the multi-level classification approach has been commonly used. However, we approached the problem as a regression task and found that using Efficientnet-B0 [10] as the backbone model resulted in high performance, as measured by the accuracy and kappa measure on the test dataset. Our approach outperformed many state-of-the-art models that were specifically designed for this task.

## 3. Approach

**Figure 3:**
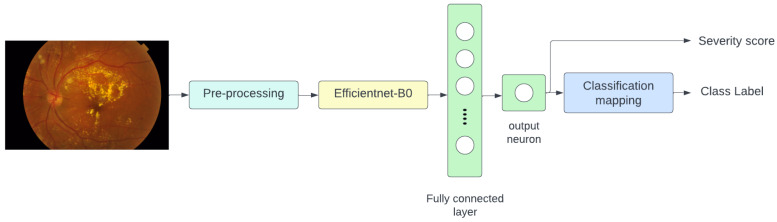
Overall architecture.

The overall architecture of the presented method is depicted in Figure 3. The input image is first subjected to a pre-processing technique that aims to normalize it and minimize the impact of variations in illumination and intra-class variation. The pre-processed image is then fed into the Efficientnet-B0 [10] model to estimate the severity of the disease. The efficientnet module generates a DR score, and the classification mapping module generates the class label based on Table 3.

### 3.1. Pre-Processing

Retinal fundus images may vary in intensity due to being collected from different sources and captured using different devices. Pre-processing can improve the generalization of a model by addressing these intensity variations. To address these variations, the images in this study were first center-cropped to remove black pixels, leaving only the fundus image portion for processing. Then, these images were resized to 256 × 256. Graham’s method [30] was then applied as a pre-processing technique to address intensity variations and improve model accuracy.

### 3.2. Efficientnet-B0 Based Regression Model

The proposed technique involves using Efficientnet-B0 [10] as the backbone for the deep learning model that is designed to classify diabetic retinopathy in retinal fundus images. Efficientnet-B0 [10] is a CNN architecture that has been shown to be effective at image classification tasks. One advantage of Efficientnet-B0 [10] compared to other CNN architectures is its efficiency in terms of the quantity of parameters and computational resources required to achieve a certain level of performance. Efficientnet-B0 [10] was designed using a combination of manual design and neural architecture search (NAS) [10], which allowed the authors to find an architecture that was both accurate and efficient.

Efficientnet-B0 is the first in a series of models designed by the authors [10] of the Efficientnet architecture. The key characteristic of Efficientnet-B0 is its efficient use of resources, with a balanced combination of depth, width, and resolution. The model has an architecture that is similar to other convolutional neural networks, with a stem, repeating blocks, and head. However, the Efficientnet-B0 architecture has been optimized for resource efficiency and performance by making use of depthwise separable convolutions; squeezed bottlenecks; and a carefully balanced combination of width, depth, and resolution. The depthwise separable convolutions allow for a reduction in computation, while the squeezed bottlenecks help to control the model’s capacity and reduce overfitting.

In terms of its structure [10], Efficientnet-B0 has an input layer followed by a series of repeated blocks of the following format:

- Squeezed bottleneck layer;
- Depthwise separable convolution layer;
- Pointwise convolution layer.

Each of these blocks is designed to be computationally efficient and to increase the model’s capacity. The model also uses a lightweight version of the MBConv block [10], which is a modified version of the standard bottleneck convolutional block used in many modern CNNs. The MBConv block reduces the number of parameters in the model, making it more computationally efficient while still maintaining high accuracy. The authors were inspired to use Efficientnet-B0 architecture for DR detection due to its advantages.

In DR classification, the disease classes are not independent, meaning that the severity of the disease is not necessarily limited to a specific set of discrete classes. Therefore, an ordinal regression model is more appropriate because it can predict a continuous severity score, allowing for a more precise prediction of disease severity. The presented approach determines a severity score and assigns a class label based on this score. A mapping table is used to determine the class label that corresponds to each severity score, as shown in Table 3. The score scale was designed with the goal of accurately reflecting the varying degrees of severity of diabetic retinopathy. The scores were assigned to each class in a way that provided meaningful differentiation between classes while also considering the limitations of the data and the complexity of the disease. This score scale was determined through multiple experiments.

### 3.3. Training Details

In this model, a transfer learning approach is utilized. The model was first trained on a large dataset for image classification and then fine-tuned on a dataset specifically for the task of classifying DR. In contrast to other DR classification models, the final layer of this model only has a single neuron, which outputs a severity score for a given image. The classification is based on this severity score. This approach allows for more accurate prediction of DR severity.

The aim of this model is to accurately predict the severity of diabetic retinopathy. To accomplish this, the mean squared error (MSE) is used as the loss function during training. The network was trained for 50 epochs using the Adam optimization algorithm [31], with a batch size of 8 and a learning rate of 1 × 10^−4^. To prevent overfitting, the training process included techniques for augmenting the data geometrically, such as scaling, flipping, and rotation. These techniques help to increase the robustness of the model and improve its generalizability to new data.

## 4. Results and Discussion

In this study, the proposed method used DDR [32], APTOS [33], and IDRiD [34] datasets. The following sections describe the characteristics of these datasets, the metrics used to assess the method’s performance, the experimental setup, the qualitative analysis, and the quantitative analysis of the results.

### 4.1. Dataset

Three datasets—DDR [32], IDRiD [34], and APTOS [33]—were utilized in this study. The characteristics of these datasets are described in the subsequent sub-sections.

#### 4.1.1. DDR

The DDR dataset, used in this study, includes 13,673 fundus images sourced from 147 hospitals located in 23 provinces in China [32]. These images have been classified into five severity categories for diabetic retinopathy: none (no-DR), mild, moderate, severe, and proliferative DR. Additionally, the dataset includes a sixth category for images with poor quality. For the purposes of this study, the images with poor quality were excluded. The class distribution of the DDR dataset is shown in Table 4. As noted in recent literature [35], the dataset is divided into three sets: 80% of the data is used for training, 10% is used for validation, and the remaining 10% is used for testing, with an equal representation of each class in each set.

#### 4.1.2. IDRiD

The IDRiD dataset comprises 516 fundus images collected from eye clinic examinations carried out by a retinal specialist in Nanded, Maharashtra, India [34]. A subset of these images has been selected for the study and includes 120 images classified as no-DR, 18 as mild, 122 as moderate, 67 as severe, and 44 as PDR. The IDRiD dataset is divided into two sets: 80% is used for training, and the remaining is used for validation, with an equal representation of each class in each set.

#### 4.1.3. APTOS

The APTOS dataset [33] is a collection of 3662 retinal images that were obtained using fundus photography at various clinics of the Aravind Eye Hospital, India. These images have been classified into five categories based on the severity of diabetic retinopathy present: no-DR (Class 0), mild NPDR (Class 1), moderate NPDR (Class 2), severe NPDR (Class 3), and PDR (Class 4). The class distribution of APTOS is shown in Table 5. The APTOS dataset, as noted in [35], is also divided into three sets: 80% is used for training, 10% is used for validation, and the remaining 10% is used for testing, with an equal representation of each class in each set.

### 4.2. Metrics

The performance of the model was evaluated using accuracy and kappa score as they are considered important measures for multi-class classification problems. Unlike other measures such as precision and recall, accuracy, and kappa score take into account the imbalanced distribution of the classes in the dataset and provide a more comprehensive assessment of the model’s performance for the classification of diabetic retinopathy.

To evaluate the effectiveness of the presented method, it is compared to other methods using two evaluation metrics: kappa score and accuracy [19]. These metrics are computed using the following equations [19].

(1) $$\mathrm{Accuracy}=\frac{\mathrm{TP}+\mathrm{TN}}{\mathrm{TP}+\mathrm{TN}+\mathrm{FP}+\mathrm{FN}}$$

In the Equation (1), TP, TN, FP, and FN refers to true positives, true negatives, false positives, and false negatives, respectively [19].

(2) $$\mathrm{Kappa}\;\mathrm{score}=1-\frac{\sum_{i=1}^{P}\sum_{j=1}^{P}w_{i,j}O_{i,j}}{\sum_{i=1}^{P}\sum_{j=1}^{P}w_{i,j}E_{i,j}}$$

In Equation (2), *p* represents the number of classes, $w_{i,j}$ represents the weight assigned to the agreement between the predicted class label and the true label for class *i* and class *j*, $O_{i,j}$ represents the observed agreement for the i, j class pair, and $E_{i,j}$ represents the expected agreement for the i, j class pair. The weight assigned to the agreement depends on how often the two classes appear together in the confusion matrix. The weights are calculated by dividing the number of instances that are labeled as both class *i* and class *j* by the total number of instances. The kappa score is a measure of the agreement between two raters, where the value of kappa ranges from −1 to 1. A value of 1 indicates perfect agreement between the two raters, while a value of −1 indicates complete disagreement. A value of 0 indicates that the agreement is no better than chance. The kappa score is often used in the context of medical diagnosis, where two or more doctors may be assessing the same patient. It can also be used in other contexts where multiple people are rating or categorizing items.

### 4.3. Quantitative Analysis

The presented model was tested on DDR [32] and APTOS datasets [33]. The model was designed to predict a DR score for each case, and the classification was determined based on this score. The classification criteria are outlined in the Table 3. The proposed method was evaluated using the accuracy and kappa score to provide a thorough assessment of its performance and to compare it to the results of previous work in the literature.

In the field of diabetic retinopathy classification, various deep learning architectures [36] have been explored for their ability to accurately predict the severity of DR from retinal images. Some of the popular deep learning architectures [36] used for this task include MobileNet, VGG, ResNet50, Inception v3, SE-ResNext, multi-scale attention network, and Efficientnet-B5. These architectures have been pre-trained on large image datasets, such as ImageNet, and then fine-tuned on the DR classification task using a technique called transfer learning.

In transfer learning, the parameters of a pre-trained network are adjusted to fit the specific task at hand, in this case DR classification. This allows the network to take advantage of the knowledge gained from the pre-training task and reduce the amount of training data required while still allowing for fine-tuning to the specific task.

The comparison of the presented model with these popular deep learning architectures was made by training each architecture using transfer learning and evaluating their performance on the APTOS and DDR test dataset. The results are explained in the following sections.

#### 4.3.1. APTOS Results

**Figure 4:**
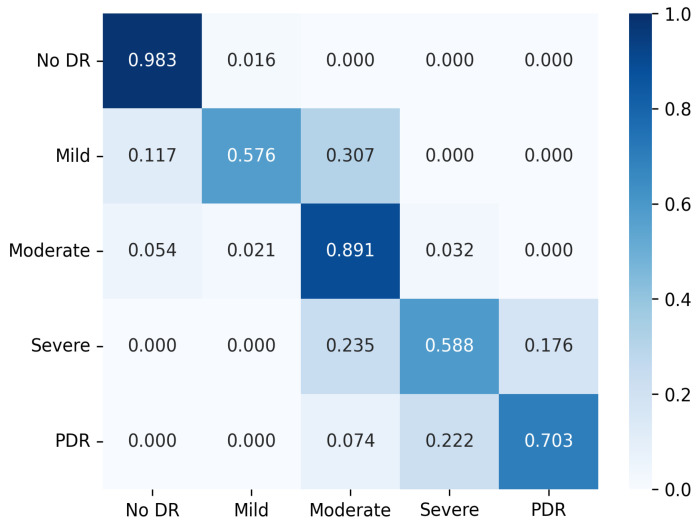
Confusion matrix for the presented method on APTOS dataset with normalization applied.

Table 6 shows the comparative results for the APTOS test dataset. As indicated in Table 6, the presented model outperformed previous literature and the top performing baseline model (SE-Resnext) in terms of accuracy, with a 2.3% improvement. The method also outperformed the multi-scale attention model. In terms of precision, the proposed method achieved a kappa score of 0.939, indicating that it is effective in classifying DR. A normalized confusion matrix is also provided to assess the model’s accuracy for each class. The normalized confusion matrix of the proposed method on APTOS dataset is shown in Figure 4. Figure 4 shows that the presented method was able to classify images without diabetic retinopathy with 98.3% accuracy. However, there was some misclassification between adjacent classes due to the close relationship between the diabetic classes.

#### 4.3.2. DDR Results

To further evaluate the effectiveness of the presented method, it was also tested on the DDR test dataset. The results of this evaluation were compared with those of previous work, using accuracy and kappa score as the evaluation metrics. Table 7 shows the comparison results.

**Figure 5:**
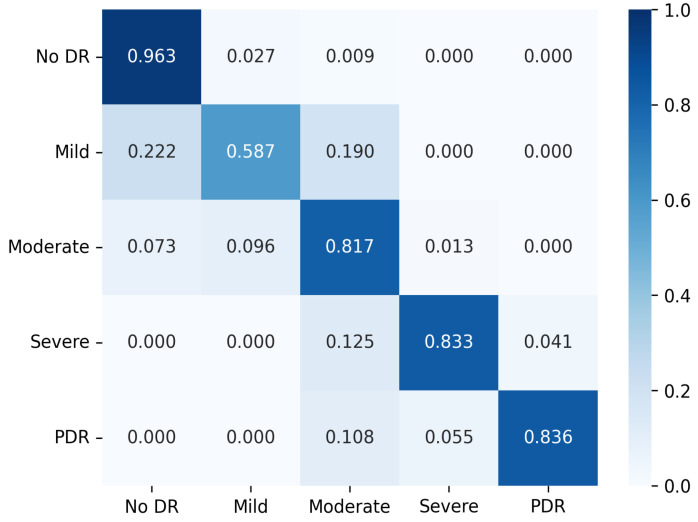
Confusion matrix for the presented method on DDR dataset with normalization applied.

The presented method demonstrated better performance in terms of accuracy and kappa score compared to other models, as shown in the Table 7. Additionally, the normalized confusion matrix in Figure 5 illustrates that the presented model effectively distinguishes between DR classes.

The presented model in the study outperformed existing state-of-the-art approaches, as evidenced by its higher accuracy and kappa score. The presented method incorporated both regression and Efficientnet-B0 architecture. This unique combination of techniques likely contributed to the improved performance of the model.

### 4.4. Qualitative Analysis

Heatmaps are graphical visualizations that use color to encode data values [37,38]. In image classification, heatmaps can be used to highlight the areas of an image that are most important for making a prediction. This can provide insight into the decision-making process of the algorithm and help identify patterns in the data. Heatmaps can also be used for qualitative analysis as they can offer a more interpretable visual representation of the data compared to raw numerical values.

**Figure 6:**
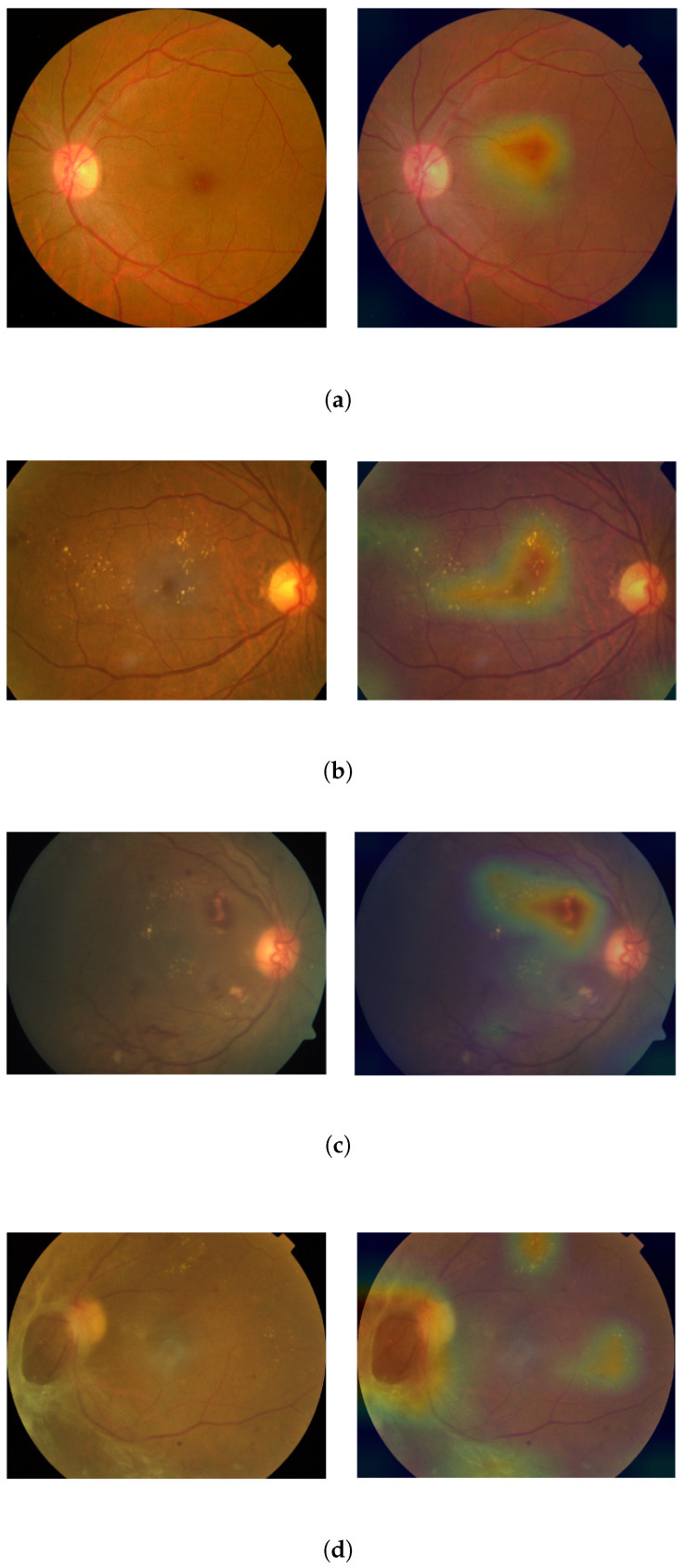
Qualitative analysis using heatmaps. (**a**) APTOS-mild NPDR. severity score: 1.12, predicted class: mild NPDR; (**b**) APTOS-moderate NPDR. severity score: 2.23, predicted class: moderate NPDR; (**c**) APTOS-severe NPDR. Severity score: 3.18, predicted class: severe NPDR; (**d**) APTOS-PDR. Severity score: 4.01, predicted class: PDR.

The presented method was evaluated qualitatively using heatmaps. Figure 6 shows the input image and its corresponding heatmap representation. The heatmap in our study is a representation of the areas of the image that have the greatest impact on the predictions made by the model. The color scale used in the heatmap, COLORMAP_JET [39], is a standard colormap used in the field of computer vision and is commonly used to visualize data that spans a large range of values. The scale ranges from blue, representing low values, to red, representing high values, with yellow in between. This allows one to effectively visualize and understand the contribution of different areas of the image to the model’s predictions. As shown in the Figure 6, the model was able to identify DR lesions and classify the image based on these lesions. Additionally, it is important to note that the red color in this figure indicates that the prediction gives more importance to that particular region. It is clear that the model was trained to focus on DR lesions when making a prediction. The DR severity score and predicted class label corresponding to each image is also depicted in Figure 6.

Additionally, heatmaps can also reveal patterns in the data that may not be immediately apparent from other forms of analysis, such as confusion matrices or accuracy scores. This information can be used to improve the model’s performance by fine-tuning the model’s hyperparameters, adding or removing layers, or modifying the data pre-processing techniques used.

### 4.5. Computational Time

Ophthalmologists spend several minutes closely examining fundus images to determine the severity of diabetic retinopathy and assign a grade [40]. This process can be challenging, especially when the patient also has other diseases. The presented method aims to automate this process and takes an average of 200 milliseconds for prediction and heatmap generation. This is significantly faster than manual diagnosis. The computational time analysis was performed on a machine with 4 cores, 28 GB of RAM, and 56 GB of disk space.

In this study, we have evaluated the computational time of the proposed model and compared it with the time required for manual diagnosis. The results showed that the proposed model significantly reduces the time required for DR prediction compared to manual diagnosis. This faster prediction time has the potential to improve the efficiency and accuracy of DR detection in real-world applications.

The reduction in the time required for DR prediction can lead to a more streamlined diagnostic process, allowing healthcare professionals to diagnose and treat the disease more quickly and effectively. This, in turn, can improve the outcomes for patients with DR by allowing for earlier detection and management of the disease.

Furthermore, the faster prediction time also has the potential to increase the accessibility of DR diagnosis, particularly in resource-limited settings where access to trained healthcare professionals is limited. This can lead to improved outcomes for patients and a reduction in the global burden of DR.

### 4.6. Discussion

A regression-based model for diabetic retinopathy classification using Efficientnet-B0 as the backbone was proposed in this study. The model was trained and tested on a dataset of retinal images, and it achieved an overall multi-class classification accuracy of 85.5% and a kappa score of 0.921 on APTOS [33] and DDR datasets [32].

The presented model demonstrated superior performance when compared to existing state-of-the-art approaches in the literature that employ alternative architectures. This can be attributed to the high efficiency and capability for superior feature extraction and classification offered by Efficientnet-B0 [10]. Furthermore, by approaching the problem of diabetic retinopathy classification through the lens of a regression problem, the accuracy of the model was enhanced. To the best of knowledge, this is the first study that views the problem of DR detection as a regression problem, which leads to improved performance and diagnostic accuracy.

A regression-based approach for DR classification has several advantages over traditional multi-class classification methods. One of the main advantages is the ability to provide a continuous output that can be interpreted as a measure of the severity of DR. This is particularly useful in cases where a quantitative measure of DR severity is needed to triage patients for further examination, such as in telemedicine systems. Additionally, the presented regression-based approaches can handle imbalanced datasets more effectively. This can lead to improved diagnostic accuracy and better patient outcomes. Furthermore, regression-based approaches can also be more robust to noise and outliers in the data as they are able to make predictions based on a broader range of information. This can be beneficial in medical imaging applications, where the quality of the images can vary widely. Overall, a regression-based approach for DR classification can provide a more accurate and comprehensive assessment of the disease and can be an important tool for the early detection and treatment of DR.

It is important to note that the model’s continuous output representing the severity of DR was mapped to class labels for the purpose of performance evaluation as the publicly available dataset used in the study had class labels and not severity scores. This mapping to class labels is simply a way to evaluate the model’s performance using the metrics and standards of traditional multi-class classification.

When compared to other studies in the field, the proposed model demonstrates superior performance in terms of accuracy, kappa score, and computational efficiency. For example, a recent study using a resnet50 architecture as the backbone achieved an accuracy of 74.6% on a APTOS dataset. Our proposed model, using Efficientnet-B0 with a regression approach, demonstrated a 11.6% increase in accuracy. Additionally, our model is computationally more efficient, which is important for real-world implementation. Moreover, the presented model is able to provide the DR severity score in addition to the class label.

The results of our study showed that the Efficientnet-B0 architecture outperformed the Efficientnet-B5 architecture in detecting diabetic retinopathy. The comparison between these two architectures highlights the trade-off between complexity and performance. The more complex architecture of Efficientnet-B5, with its larger number of parameters, resulted in overfitting to the training data. Additionally, the larger size of Efficientnet-B5 made it more computationally intensive, which led to longer training times and potentially impacted its performance. Furthermore, it is possible that the more complex architecture of Efficientnet-B5 was not well-suited for the specific task of diagnosing diabetic retinopathy and the simpler architecture of Efficientnet-B0 was more effective in this context. These findings emphasize the importance of considering the architecture of a model when selecting it for a specific task.

The results of this study have important implications for the early detection and treatment of DR. The proposed model can be used as a reliable tool for screening patients in resource-limited settings, where access to ophthalmologists is limited. Additionally, the model can be easily integrated into telemedicine systems, which can aid in the early detection and treatment of DR in remote areas.

The key findings of the study demonstrate the potential for a regression-based approach, incorporating the Efficientnet-B0 architecture to effectively classify and predict the severity of diabetic retinopathy. This approach provides a more interpretable way to predict DR compared to traditional classification methods.

However, it is important to acknowledge that this approach also has limitations that could impact its performance. The limitations of this study include the difficulty in fully incorporating the inter-relationships between different severity levels into the model, and the fact that the results are based on a specific dataset, requiring further investigation to assess the generalizability of the proposed model to other datasets.

Future research could focus on exploring alternative regression models and architectures to improve performance and incorporating additional sources of information, such as patient medical history, to enhance accuracy. Additionally, the integration of advanced machine learning techniques, such as transfer learning or ensemble methods, could be explored to further optimize the model’s performance.

## 5. Conclusions

In this paper, we present an Efficientnet-B0-based CNN model for the classification of diabetic retinopathy. Our method effectively extracts non-handcrafted features for classification and outperforms classical algorithms, as demonstrated by our experimental analysis. Training our model using a regression approach leads to improved performance when compared to other methods. The proposed method is also computationally efficient, making it suitable for real-time applications.

The proposed regression-based model, utilizing Efficientnet-B0 as the backbone for DR classification, demonstrates promising performance and computational efficiency. It has the potential to be a useful tool for the early detection and treatment of DR in resource-limited settings and telemedicine systems. With strong potential for clinical use in the future, we plan to continue improving our results by employing multiple models for feature extraction in future work. Additionally, future research should focus on evaluating the model’s performance on other datasets and incorporating more complex architectures to improve its performance in more severe cases of DR.

## Figures and Tables

**Table 1:** DR progression stages.

Disease Severity Level	Findings
Grade 0: No DR	No visible signs of the disease
Grade 1: Mild NPDR	Only small, round dots on the retina (microaneurysms) are present
Grade 2: Moderate NPDR	There are more than just microaneurysms present, but the disease is not yet at the severe NPDR stage
Grade 3: Severe NPDR	The presence of any of the following symptoms, with no evidence of proliferative diabetic retinopathy: more than 20 intraretinal haemorrhages in each of four quadrants, definite venous beading in two or more quadrants, or prominent intraretinal microvascular abnormalities (IrMA) in one or more quadrants
Grade 4: Proliferative DR	The presence of either neovascularization or vitreous/preretinal haemorrhages, or both

**Table 2:** Overview of studies on deep learning-based diabetic retinopathy classification.

Reference	Architecture/Model	Research Concept
Nneji et al., 2022 [18]	Inception V3	An artificial neural network that combines multiple deep learning techniques and uses weighting to extract features and classify different stages of DR has been created
Mohammad D. Alahmadi [19]	Inception encoder with attention block	Proposed style and content recalibration mechanism inside the deep neural network
Huang et al., 2022 [20]	ResNet50	Transfer learning using ResNet50 as backbone
Deepa et l., 2022 [21]	InceptionV3, Xception	An ensemble of deep-CNN is implemented for the DR classification
Tariq et al., 2021 [22]	AlexNet, GoogleNet, Inception V4, Inception resnetv2, and ResNext-50	Using pre-trained models with custom dataset
Devi et al., 2021 [23]	DenseNets	Utilizing the deep features from multiple convolution blocks of a pre-trained model
Reguant et al., 2021 [24]	inception, ResNet50, Inception ResNet, Xception, and Goal of this study is to uncover the inherent features of images and their clinical significance	
Dai et al., 2021 [25]	DeepDR	Developed DL system to detect DR stages
Bhardwaj et al., 2021 [26]	AlexNet, GoogleNet, resNet, VGG16, VGG19, Inception v3	CNN for feature extraction and SVM is used for classification
Yaqoob et al., 2021 [27]	ResNet50, random forest	ResNet50 is employed for feature extraction and random forest is used as the classifier
Majumder and Kehtarnavaz, 2021 [11]	DenseNet	Developed multitask model
Islam et al., 2021 [28]	DiaNet	Developed a multi stage classifier using DiaNet
Riaz et al., 2020 [29]	DenseNets	Utilizing deep, densely connected networks for retinal image analysis, with training starting from scratch

**Table 3:** Classification criteria.

Score	Classlabel
Score <0.5	No-DR
0.5≤ Score <1.5	Mild NPDR
1.5≤ Score <2.5	Moderate NPDR
2.5≤ Score <3.5	Severe NPDR
Score ≥3.5	PDR

**Table 4:** Distribution of classes in the DDR dataset [32].

Label	Count
No DR	5639
Mild NPDR	567
Moderate NPDR	4029
Severe NPDR	212
Proliferative DR	822

**Table 5:** Distribution of classes in the APTOS dataset [33].

Label	Count
No DR	1805
Mild NPDR	370
Moderate NPDR	999
Severe NPDR	193
Proliferative DR	295

**Table 6:** Quantitative analysis—APTOS.

Model	Accuracy	Kappa Score
Inception v3	0.788	0.822
MobileNet	0.792	0.832
VGG	0.812	0.887
ResNet50	0.746	0.801
Efficientnet-B5	0.825	0.906
SE-ResNext	0.839	0.913
Multi-scale attention	0.846	0.896
Presented Method	0.862	0.939

**Table 7:** Quantitative analysis—DDR.

Model	Accuracy	Kappa Score
Inception v3	0.758	0.792
MobileNet	0.752	0.800
VGG	0.798	0.857
ResNet50	0.721	0.790
Efficientnet-B5	0.823	0.865
SE-ResNext	0.813	0.878
Multi-scale attention	0.815	0.869
Presented Method	0.848	0.903

## Data Availability

The presented method uses three publicly available datasets: APTOS, DDR, and IDRiD. The URLs for accessing these datasets are (1) APTOS: https://www.kaggle.com/c/aptos2019-blindness-detection/data accessed on 1 August 2021; (2) DDR: https://github.com/nkicsl/DDR-dataset accessed on 20 July 2021; and (3) IDRiD: https://idrid.grand-challenge.org/ accessed on 1 August 2021.

## Abbreviations

The following abbreviations are used in this manuscript:

DR	Diabetic retinopathy
CNN	Convolutional neural network
NPDR	Non-proliferative diabetic retinopathy
PDR	Proliferative diabetic retinopathy
ICDRDSS	International clinical diabetic retinopathy disease severity scale
IrMA	Intraretinal microvascular abnormalities
ADTCWT	Anisotropic dual-tree complex wavelet transform
NAS	Neural architecture search
MSE	Mean squared error
TP	True positive
TN	True negative
FP	False positive
FN	False negative
